# Myasthenia Gravis and its Association With Thyroid Diseases

**DOI:** 10.7759/cureus.10248

**Published:** 2020-09-04

**Authors:** Saba Amin, Myat Aung, Fenil R Gandhi, Julio A Pena Escobar, Azouba Gulraiz, Bilal Haider Malik

**Affiliations:** 1 Medicine, California Institute of Behavioral Neurosciences & Psychology, Fairfield, USA; 2 Internal Medicine, California Institute of Behavioral Neurosciences & Psychology, Fairfield, USA

**Keywords:** myasthenia gravis (mg), myasthenia gravis and thyroid disease, mg and graves disease, mg and autoimmune diseases, autoimmune disease, myopathy, hashimoto's thyroiditis, thyroid carcinoma, thymectomy, myasthenia crisis

## Abstract

Myasthenia gravis (MG) is a rare autoimmune neuromuscular junction disorder, and thyroid disorder is a disorder involving the thyroid receptor, of which Graves' disease (GD) is the most common autoimmune thyroid disorder, in which antibodies develop against thyroid receptors. Both may have similar clinical features. In myasthenia gravis, autoimmune antibodies develop against postsynaptic neuromuscular junction disrupting the neuromuscular transmission, resulting in fluctuating muscle weakness and fatigue. It is a disease of young women and older men. The two pathologies may coexist in a patient or can precede one another. Graves' disease (GD) among thyroid diseases is most often associated with MG.

Similarities in clinical features lead to difficulty in distinguishing MG and GD. Despite the standard treatment of myasthenia gravis, including steroids, acetylcholinesterases, rituximab, immunosuppressants, and thymectomy, there is still an increased number of relapses and myasthenia crisis. Eculizumab and plasmapheresis are the two new treatment options for MG, with supporting evidence of marked improvement in recent studies. Myasthenia gravis and Graves' disease have a see-saw relationship. Treating one pathology may worsen the other, so physicians should always consider MG as a differential in patients of hyperthyroidism presenting with new symptoms of fatigue or respiratory failure or neuromuscular weakness.

In this comprehensive review article, we tried to establish an association between myasthenia gravis and Graves' disease (GD) by exploring currently available literature from PubMed. However, more studies need to be done to establish an association between pathologies.

## Introduction and background

Specific immune responses directed against the structures of the self components result in autoimmune diseases (AID). Likewise, autoimmune thyroid disease (ATD) results in autoimmunity against thyroid antigens, and it comprises two diseases: Graves' disease and Hashimoto's thyroiditis. Myasthenia gravis (MG) is related to other AID, such as autoimmune thyroid disease. Both of these pathologies share some standard features like pathophysiological mechanisms and genetic predisposition, yet they also have some differences like in treatment modalities [1].

Myasthenia gravis is an autoimmune pathology that involves postsynaptic neuromuscular junction. It is defined as the development of specific antibodies against postsynaptic acetylcholine receptors mostly and to a lesser extent against muscle-specific kinase protein resulting in fluctuating muscle weakness. Most of the patients present with ptosis and diplopia initially and then progress rapidly to generalized disease. It causes acute respiratory failure in patients, and a distinct feature of this respiratory failure is its mediation by the neurologic process [2]. MG is more prevalent in women, and its incidence is reported more in the elderly. There are several diagnostic tools available with varying sensitivity and specificity for MG like ice pack test, neostigmine test, electrophysiological studies, rest test, and circulating antibody measurement. The treatment options for myasthenia gravis are acetylcholinesterase inhibitors, corticosteroids, immunosuppressants, and thymectomy which can slow down its progression to become generalized disease [3]. However, most of the time, the standard therapy for MG is not so beneficial because of its late onset of action and fails in achieving remission of the disease. In recent studies, a monoclonal antibody against CD20 B-cell surface receptors named rituximab (RTX) has shown efficacy in treating MG in patients refractory to standardized treatment [4].

Graves' disease (GD) and MG are autoimmune diseases, and their coexistence reported in many patients. In GD, there is the development of autoantibodies against thyroid-stimulating hormone receptors resulting in thyrotoxicosis. Other clinical features are ophthalmopathy, dermopathy, and acropachy. Sometimes MG diagnosis is missed in routine clinical practice due to its subtle manifestations. Ocular manifestations' similarities make it difficult for physicians to differentiate; therefore, high vigilance needed in making the diagnosis [5].

See-saw relationship between these two pathologies, MG and GD, has been reported in the past by some authors. Treating one pathology may worsen the other which will make it a challenge to treat both pathologies. Myasthenia gravis gets worse by the use of antithyroid drugs through immunomodulatory effects. Beta-blockers and corticosteroids cause a worsening of weakness in myasthenia patients [6].

Cross-reaction between thyroid receptors and the neuromuscular junction immunologically is seen in patients of MG and GD. Among autoimmune thyroid disorders, hyperthyroidism association with MG is most common [7]. We should always look for the concomitant association of MG in patients with autoimmune diseases who present with fatigue, newly developed respiratory failure, or neuromuscular weakness [8].

## Review

Myasthenia gravis (MG) and Graves' disease are well known autoimmune diseases, and their coexistence in the same individual is seen often [9]. MG is more prevalent in the female population than the male [3]. MG clinical features include fatigue and fluctuating muscle weakness of different muscle groups of the body. Myasthenia gravis is also associated with other autoimmune diseases but most often associated with autoimmune thyroid disease (ATD) [9]. Both pathologies share the same pathophysiological mechanism along with similarities in clinical features, e.g., muscular weakness. Thus, it leads to diagnostic confusion in individuals having both diseases [9]. With the advancement in therapeutic options for MG, we came to know about monoclonal antibody eculizumab and plasmapheresis as effective treatment modalities for MG. Graves' disease (GD) is most commonly associated with MG among all autoimmune thyroid diseases [5]. Sometimes we need to do more laboratory investigations to distinguish one pathology from another. Besides standard symptomatic treatment for both pathologies, there is another issue where treating one pathology may worsen the other. MG is also associated with other immune and non-immune pathologies.

Myasthenia gravis

Myasthenia gravis is an autoimmune disease caused by antibodies against postsynaptic neuromuscular junction components [10]. It is characterized by fluctuating muscle weakness and fatigue of different muscle groups of the body, including ocular, bulbar, and skeletal muscles of proximal limbs. It can also present as a paraneoplastic syndrome in the form of a thymic tumor. The immunologic mechanism behind MG involves impairment of peripheral self-tolerance and central thymic mechanisms resulting in B-cells activation, mediated by CD4 T-cells leading to pathogenic antibodies of IgG subclass. These antibodies will bind to either of these four proteins, acetylcholine receptor (AchR), or muscle-specific kinase (MuSK), lipoprotein receptor protein 4 (LRP4), and agrin disrupting the neuromuscular transmission resulting in clinical features of the disease [11].

Incidence and prevalence

An increase in the incidence rate and prevalence of MG seen in the recent past, specifically in the elderly population [12]. The prevalence of MG in the world ranges from 50/1,000,000 in Hong Kong to 200/1,000,000 in Virginia (USA), and the annual incidence rate ranges between 0.25 to 20 per 1,000,000 population. MG onset is between 20-40 years of age, and women comprise 60% of this group [13]. A large US cohort study from 2000 to 2005, including 5,502 patients for evaluating incidence and mortality rates in the USA, was done [14]. Men show a higher incidence rate in their sixth, seventh, and eighth decades while women show a higher incidence rate during the first five decades. The mortality rate was 2.2% in the hospital. So it is a disease of older men and young women [14]. A national study in Taiwan showed a high incidence rate in the elderly population, and prevalence was 140 per million. The men-to-women ratio was 0.7 [3].

Diagnosis and treatment of myasthenia gravis

There are several diagnostic tools available with varying sensitivity and specificity for MG like ice pack test, neostigmine test, electrophysiological studies, rest test, and circulating antibody measurement [3]. Despite advances in the understanding of treatment modalities and pathophysiology, still an increasing number of MG exacerbations within one to two years of diagnosis. The morbidity rate is also higher [15]. MG's beneficial treatment options are steroids, acetylcholinesterase inhibitors, immunosuppressive agents, azathioprine, cyclosporine, cyclophosphamide, mycophenolate mofetil, tacrolimus, etanercept, intravenous immunoglobulin, and thymectomy [15]. Besides acetylcholinesterase inhibitors, thymectomy has proven efficacy in thymomatous and non-thymomatous MG [16].

Treatment advances for MG

Plasmapheresis and eculizumab have been approved by US food and drug administration in the recent past for MG [16]. Since MG is an antibody-mediated disorder that leads to B-cells activation and thus complement activation, its treatment mainly focuses on removing these auto-antibodies and complement inhibition [17]. We have different approaches to gain this goal like plasmapheresis and complement inhibitor, eculizumab [17]. Plasmapheresis indicated in the case of myasthenic crisis and has three ways: plasma exchange, double filtration plasmapheresis, and immunoadsorption [18]. Eculizumab reported in AchR antibodies positive individuals in the USA with generalized MG and for refractory MG. It has also shown efficacy in patients in Japan who show no benefit to IV immunoglobulin and plasma exchange [19]. Because of the advances in interventions for managing MG, the mortality rate has decreased in past decades. In the last two to three years, the standard therapy for MG has changed a lot [20].

Myasthenia gravis and other autoimmune diseases

Since MG is an autoimmune disease, other autoimmune diseases (AID) can co-occur with MG. Among all autoimmune diseases, autoimmune thyroid diseases are most commonly seen with MG, and it can impact its clinical course [21]. MG has two types based on the presence or absence of acetylcholine receptor antibodies (AchR antibodies), those with AchR antibodies are seropositive, and those without it are seronegative. Seropositive MG patients linked with other endocrinological disorders [22]. MG association with other diseases is established by doing a study on a group of 127 people and followed them for ten years. The outcome of the survey showed onset of disease before age 50, more prevalent in women, 65% of patients showed another condition, and 15% had an autoimmune disease. Associated diseases are systemic lupus erythematosus (SLE), hypertension, heart disease, and dyslipidemia. Around 10.2% were diagnosed with extrathymic tumors [23]. 

A retrospective study states the relation between MG and endocrinological disorders. One hundred and nine patients of MG participated and distinguished based on AchR antibodies presence or absence. Seropositive MG patients were 66% in the study with thymic hyperplasia, thymoma, or thyroid disease [24]. Another retrospective study was done from 2008 to 2009 on 300 patients of MG for linkage of MG with thyroid disease. Study results showed that MG patients with positive thyroid antibodies have a higher number of AchR antibodies and more abnormalities with T cells [25]. Of all the thyroid diseases, Graves' disease is more frequent in patients with MG. Clinical features like neuromuscular weakness and ocular signs cause diagnostic confusion between two pathologies [24]. 

A patient with thyrotoxicosis presents with fatigue initially. While managing his thyrotoxicosis, he experienced severe respiratory failure. This respiratory failure resulted after giving medication for his thyrotoxicosis, which unmasked the myasthenia gravis. Physicians should also consider MG in hyperthyroid patients presenting with neuromuscular weakness [25]. So, there is diagnostic confusion between MG and Graves' disease because of similarities in clinical features, and co-occurrence of these two autoimmune pathologies seen often in the same individual. MG pathology involves thymus, i.e., thymic hyperplasia and thymoma seen in early-onset and late-onset MG, respectively. Graves' disease pathology also includes the thymus, i.e., thymic hyperplasia and thymus lymphoid hyperplasia. There is a case report of Graves' disease, which shows myasthenia like symptoms and thymic enlargement on imaging, but diagnostic testing for MG was negative, and treatment of Graves' disease leads to complete resolution of symptoms and thymic enlargement [26]. Because of diagnostic confusion, there are also different approaches in treating MG with and without thyroid disease, and treatment of one pathology may worsen the other one. A literature study presented a patient with exophthalmos, right-sided lid retraction, and diplopia. Graves' disease is confirmed after running lab investigations with TSH antibodies. Symptoms improved with antithyroid medications and steroids but came back two weeks later with new left-sided ptosis and diplopia recurrence. Diagnostic testing for MG came back positive and diplopia and ptosis improved with pyridostigmine. So, while managing Graves' ophthalmopathy, we should also consider MG [27].

Treating MG in the hyperthyroid patient is sometimes challenging as there was a report of three cases of MG patients with hyperthyroidism treated with pyridostigmine and beta-blockers leading to improvement in hyperthyroid symptoms. However, myasthenic symptoms get worsened in two patients and no increase in one patient. As thymic hyperplasia seen in both pathologies, thymectomy leads to improvement in all three patients [28]. So, treating hyperthyroidism may worsen MG symptoms in the same individual as antithyroid drugs through their immunomodulatory mechanism may worsen the MG symptoms [29].

Thyroid disease prevalence and risk in MG patients

The prevalence and risk of thyroid diseases in MG are higher than in the general population. Both pathologies being autoimmune diseases may be the reason for their coexistence in the same person. The literature review showed a higher prevalence and a higher risk of thyroid diseases in MG patients, during the follow-up period, after the diagnosis compared to the general population.

The study includes 5,813 MG patients in the case group and 29,065 non-MG in the control group. Thyroid disease prevalence in myasthenia gravis individuals was 18.4% (odds ratio [OR] 3.895, 95% confidence interval [CI] {3.574-4.246}); comorbid thyroid disease incidence was 8.7% in the case group and 4% in the control group.

The myasthenia gravis patients had a 2.36-fold increased risk of developing thyroid diseases compared to the non-MG people (crude hazard ratio [HR] 2.360, 95% CI {2.095-2.659}). The cumulative probability of developing thyroid disease at 1, 5, and 10 years was 21.6%, 24.9%, and 28.7%, respectively, in the case group, while the cumulative probability was 6.5%, 8.8%, and 11.8% respectively in the control group (log-rank test <0.0001) [30].

Hashimoto's thyroiditis and MG

Hashimoto's thyroiditis co-occurrence in MG patients is rare but well-known. Graves disease is seen more often with myasthenia gravis among thyroid diseases, but Hashimoto's thyroiditis presence in people with myasthenia gravis has also seen in recent studies. 

Krol did a study in which he presented patients with a history of myasthenia gravis presented with increasing bulbar and proximal muscle weakness. Appropriate investigations like neck and chest imaging, lab investigations, and blood workup were done to test the cause. Hashimoto's thyroiditis' diagnosis is supported by the presence of thyroid antibodies, diffuse thyroid enlargement, increased thyroid-stimulating hormone (TSH) , and decreased T2 and T4 levels [31]. Moreover, Hashimoto et al. studied the association of Hashimoto's thyroiditis with MG [32]. They presented the case of individuals diagnosed with MG established clinically by pharmacologic tests, electromyography, and the presence of anti-AchR antibodies. The coexistence of Hashimoto's thyroiditis diagnosed by the presence of diffuse thyroid enlargement, anti-thyroglobulin plus anti-microsomal antibodies, and lower 131I uptake. Pyridostigmine and ephedrine chloride was given as a treatment, but the delusion of persecution developed as a result. The symptoms vanished after the discontinuation of ephedrine chloride. The authors concluded that Hashimoto's thyroiditis could be present in myasthenia gravis patients but rarely [32]. 

Thyroid carcinoma and MG

Apart from thyroid diseases, thyroid carcinoma is also associated with myasthenia gravis. Cancer presence in MG patients is rare but seen in several literature studies.

Senga et al. did a study and found the rare presence of thyroid carcinoma and myasthenia gravis at one time in individuals [33]. One of the cases presented with proximal muscle weakness and ptosis of the right eye diagnosed with myasthenia gravis after running pharmacologic tests and antibody tests. Neck and chest imaging showed the presence of thymoma and planned to do thymectomy. After the thymectomy, the patient complained of swelling in the neck. Upon examination, the swelling of several cervical lymph nodes was evident. Biopsy of lymph nodes showed metastatic thyroid carcinoma. Subsequently, thyroid carcinoma with metastasis to the cervical lymph node, the diagnosis was made, and total thyroidectomy with cervical lymph nodes removal planned. Total thyroidectomy with no post-operative complications was done. Radical dissection of bilateral cervical lymph nodes was done 37 days after the total thyroidectomy; no post-operative complication or recurrence happened at that time. So, in patients of MG with occult thyroid carcinoma, a different treatment modality should be used. For better and easier post-operative management, a two-stage operation is better than one-stage. It is concluded from the studies that while managing myasthenia gravis associated with thymoma, we should also consider thyroid diseases and thyroid carcinoma as a differential diagnosis [33]. The following table shows the epidemiology, pathology, and treatment options for autoimmune thyroid diseases (ATD) and myasthenia gravis (MG) (Table 1) [34-39].

**Table 1:** Epidemiology, Pathology, and Treatment Options for ATD and MG MG: myasthenia gravis; AchR: acetylcholine receptor; TSHR: thyroid-stimulating hormone receptor; TPO: thyroid peroxidase; MuSK: muscle-specific kinase; ATD: autoimmune thyroid diseases

	Graves' disease	Hashimoto’s hypothyroidism	AchR-MG	MuSk-MG
Incidence	2%	2%	0.1%	0.01%
Prevalence	10%	10%	3%	6%
Pathology	Thyroid hyperplasia	Thyroid destruction	Thymus hyperplasia or thymoma	No thymic involvement
Target of autoantibody	TSHR	TPO (90-95%) TG (20-50%)	AchR	MuSK
Diagnostic test	Physical exam, blood sample, radioactive iodine uptake	Physical exam, blood sample,	Electromyography, Tensilon test	Blood test
Treatment	Antithyroid drugs, radioactive iodine, surgery	Levothyroxine	Anticholinesterase, steroids, eculizumab, thymectomy	Steroids, rituximab

## Conclusions

The prevalence of myasthenia is more in the female and elderly population. However, despite advancements in understanding pathology and treatment strategy, there is still an increased number of myasthenic crisis episodes. The relationship between MG and Graves' diseases is controversial even now.

Similarities in clinical features among both pathologies have resulted in not detecting the other autoimmune disease, which then appears as an unusual outcome, disease severity, and treatment failure. Furthermore, treating thyroid disease sometimes leads to worsening of myasthenia gravis symptoms. Further studies and research needed to discover new distinguishing tests for both pathologies. More research is required to clarify the association and yield a better management strategy so that we have a better guideline to follow. Moreover, we should do case-control studies, case series, and randomized clinical trials as we do not have much data available at the moment, to get more knowledge about the association between these two pathologies and devise a better management plan.

## References

[1] Lopomo A, Berrih-Aknin S (2017). Autoimmune thyroiditis and myasthenia gravis. Front Endocrinol.

[2] Kozak HH, Uca AU, Teke T, Altas M, Karatas E (2016). Myasthenia gravis with acute respiratory failure in the emergency department. Turk J Emerg Med.

[3] Lin CW, Chen TC, Jou JR, Woung LC (2018). Update on ocular myasthenia gravis in Taiwan. Taiwan J Ophthalmol.

[4] Iorio R, Damato V, Alboini PE, Evoli A (2015). Efficacy and safety of rituximab for myasthenia gravis: a systematic review and meta-analysis. J Neurol.

[5] Mangaraj S, Choudhury AK, Mohanty BK, Baliarsinha AK (2016). Neurological manifestations of Graves' disease: a case report and review of the literature. J Neurosci Rural Pract.

[6] Mallikarjuna SK, Velayutham SS, Sowmini PR, Jeyaraj MK, Arunan S (2019). See-saw relationship and its reversal after immunotherapy in a case of Graves' disease with coexisting myasthenia gravis. J Neurosci Rural Pract.

[7] Salhi H, Ajdi F (2019). Hypothyroidism and myasthenia: a case study. Pan Afr Med J.

[8] Nacu A, Andersen JB, Lisnic V, Owe JF, Gilhus NE (2015). Complicating autoimmune diseases in myasthenia gravis: a review. Autoimmunity.

[9] Masood I, Yasir M, Aiman A, Kudyar RP (2009). Autoimmune thyroid disease with myasthenia gravis in a 28-year-old male: a case report. Cases J.

[10] Berrih-Aknin S, Frenkian-Cuvelier M, Eymard B (2014). Diagnostic and clinical classification of autoimmune myasthenia gravis. J Autoimmun.

[11] Melzer N, Ruck T, Fuhr P (2016). Clinical features, pathogenesis, and treatment of myasthenia gravis: a supplement to the Guidelines of the German Neurological Society. J Neurol.

[12] Binks S, Vincent A, Palace J (2016). Myasthenia gravis: a clinical-immunological update. J Neurol.

[13] Pekmezović T, Lavrnić D, Jarebinski M, Apostolski S (2006). Epidemiology of myasthenia gravis. Srp Arh Celok Lek.

[14] Alshekhlee A, Miles JD, Katirji B, Preston DC, Kaminski HJ (2009). Incidence and mortality rates of myasthenia gravis and myasthenic crisis in US hospitals. Neurology.

[15] García-Carrasco M, Escárcega RO, Fuentes-Alexandro S, Riebeling C, Cervera R (2007). Therapeutic options in autoimmune myasthenia gravis. Autoimmun Rev.

[16] Farmakidis C, Pasnoor M, Dimachkie MM, Barohn RJ (2018). Treatment of myasthenia gravis. Neurol Clin.

[17] Wang S, Breskovska I, Gandhy S, Punga AR, Guptill JT, Kaminski HJ (2018). Advances in autoimmune myasthenia gravis management. Expert Rev Neurother.

[18] Konishi T (2008). Plasmapheresis in patients with myasthenia gravis. Nihon Rinsho.

[19] Dhillon S (2020). Eculizumab: a review in generalized myasthenia gravis. Drugs.

[20] Jordan A, Freimer M (2018). Recent advances in understanding and managing myasthenia gravis. F1000Res.

[21] Kubiszewska J, Szyluk B, Szczudlik P (2016). Prevalence and impact of autoimmune thyroid disease on myasthenia gravis course. Brain Behav.

[22] Toth C, McDonald D, Oger J, Brownell K (2006). Acetylcholine receptor antibodies in myasthenia gravis are associated with greater risk of diabetes and thyroid disease. Acta Neurol Scand.

[23] Tanovska N, Novotni G, Sazdova-Burneska S (2018). Myasthenia gravis and associated diseases. Open Access Maced J Med Sci.

[24] Chen YP, Wei DN, Chen B (2010). The clinical features of myasthenia gravis associated with thyroid abnormalities. Zhonghua Nei Ke Za Zhi.

[25] Sehgal S, Rebello R, Wolmarans L, Elston M (2017). Hickam's dictum: myasthenia gravis presenting concurrently with Graves' disease. BMJ Case Rep.

[26] Lakhal K, Blel Y, Fysekidis M, Mohammedi K, Bouadma L (2008). Concurrent Graves disease thyrotoxicosis and myasthenia gravis: the treatment of the former may dangerously reveal the latter. Anaesthesia.

[27] Nicolle MW (1999). Pseudo-myasthenia gravis and thymic hyperplasia in Graves' disease. Can J Neurol Sci.

[28] Widjaja A, Rademaker J, Gölkel C, Holstein A, Leifke E, Wat N (2000). Basedow-ophthalmopathie und okuläre myasthenie [Graves ophthalmopathy and ocular myasthenia]. Ophthalmologe.

[29] Teoh R, Chow CC, Kay R, Cockram CS, McGuire L (1990). Response to control of hyperthyroidism in patients with myasthenia gravis and thyrotoxicosis. Br J Clin Pract.

[30] Chou CC, Huang MH, Lan WC, Kong SS, Kuo CF, Chou IJ (2020). Prevalence and risk of thyroid diseases in myasthenia gravis [PREPRINT]. Acta Neurol Scand.

[31] Krol TC (1979). Myasthenia gravis, pernicious anemia, and Hashimoto's thyroiditis. Arch Neurol.

[32] Hashimoto A, Hanada M, Okada S, Aoki N (1981). A case report of myasthenia gravis associated with Hashimoto's thyroiditis. Folia Psychiatr Neurol Jpn.

[33] Senga O, Hikita H, Kinoshita T, Hara K, Miyakawa M (1992). Myasthenia gravis with thymoma associated with occult thyroid carcinoma. Surg Today.

[34] Topliss DJ (2016). Clinical update in aspects of the management of autoimmune thyroid diseases. Endocrinol Metab.

[35] Drachman DB (2016). Myasthenia gravis. Semin Neurol.

[36] Dong YH, Fu DG (2014). Autoimmune thyroid disease: mechanism, genetics, and current knowledge. Eur Rev Med Pharmacol Sci.

[37] Gilhus NE, Skeie GO, Romi F, Lazaridis K, Zisimopoulou P, Tzartos S (2016). Myasthenia gravis—autoantibody characteristics and their implications for therapy. Nat Rev Neurol.

[38] Jacobson DL, Gange SJ, Rose NR, Graham NM (1997). Epidemiology and estimated population burden of selected autoimmune diseases in the United States. Clin Immunol Immunopathol.

[39] Antonelli A, Ferrari SM, Corrado A, Di Domenicantonio A, Fallahi P (2015). Autoimmune thyroid disorders. Autoimmun Rev.

